# Empirical parameterisation and dynamical analysis of the allometric Rosenzweig-MacArthur equations

**DOI:** 10.1371/journal.pone.0279838

**Published:** 2023-02-27

**Authors:** Jody C. McKerral, Maria Kleshnina, Vladimir Ejov, Louise Bartle, James G. Mitchell, Jerzy A. Filar

**Affiliations:** 1 College of Science and Engineering, Flinders University, Adelaide, SA, Australia; 2 Institute of Science and Technology, Klosterneuburg, Austria; 3 Department of Microbiology and Infectious Diseases, Université de Sherbrooke, Sherbrooke, Québec, Canada; 4 School of Mathematics and Physics, The University of Queensland, St Lucia, QLD, Australia; National Cheng Kung University, TAIWAN

## Abstract

Allometric settings of population dynamics models are appealing due to their parsimonious nature and broad utility when studying system level effects. Here, we parameterise the size-scaled Rosenzweig-MacArthur differential equations to eliminate prey-mass dependency, facilitating an in depth analytic study of the equations which incorporates scaling parameters’ contributions to coexistence. We define the functional response term to match empirical findings, and examine situations where metabolic theory derivations and observation diverge. The dynamical properties of the Rosenzweig-MacArthur system, encompassing the distribution of size-abundance equilibria, the scaling of period and amplitude of population cycling, and relationships between predator and prey abundances, are consistent with empirical observation. Our parameterisation is an accurate minimal model across 15+ orders of mass magnitude.

## Introduction

Allometric scaling relationships have been the subject of scrutiny and debate since the connection between organism size and its metabolic rate was first defined by Rubner in 1883 [1–4]. These models, which link some characteristic *y* to the size *x* of an organism via the power law *y* = *ax*^*b*^ (where *a*, *b* are scalar constants), are appealing due to their capacity to capture a multitude of relationships despite their simplicity. Scaling laws have been used to express a variety of biological rate measures, such as metabolism, consumption, and birth or death rates [5–8]. Allometry is also utilised in modelling behavioural traits and bioenergetic characteristics, such as movement behaviour or locomotory costs [7, 9–11]. At broad scales, such laws have been applied to ecosystem-level properties, including predictions of organism population density and carrying capacity [12–14]. However, despite scaling laws’ wide utility and intensive study, there has been a limited exploration of the properties of minimally constructed, size-generalised predator-prey models [15–17].

Many authors have examined the empirical relationship between organism and population sizes [12, 13, 18–20]. Reported exponents fall between −1 and −1/4 depending on factors such as taxonomy or environment. The classical −3/4 value describing global size-density relationships is the direct inverse of Kleiber’s 3/4 law for metabolic scaling [2, 13], leading to the ‘energetic equivalence’ hypothesis: that is, the net energy contained within each size class is invariant [18, 20]. This conjecture has been widely debated, particularly with respect to whether this invariance is cause or effect of other bioenergetic drivers [21]. However, despite disagreement over underlying mechanisms, there is broad consensus that the consistency of size-density scaling within empirical data likely reflects fundamental physical constraints [5, 21]. To examine what drives limitations in macro-scaling behaviour, it is possible to use dynamical size-based models incorporating organism traits that scale across the size range [6, 15, 16]. This approach facilitates the investigation of critical breaks in ecosystem-level scaling laws within a global framework, and the exploration of potential impacts from changes that may affect many organisms in a similar way—for example, warming temperatures or emergent hypoxia in the oceans [22, 23]. However, perturbing parameters across 15+ orders of magnitude in size poses challenges. For example, coexistence regions of size-generalised predator-prey models are dominated by scaling exponents [16].

It is more straightforward to keep model behaviour stable, thus resolving the coexistence issue, by using the 4-parameter Lotka-Volterra model, but that setting is too simple for some applications [6, 17]. Alternately, a series of models may be solved piece-wise for different size classes, yet this means that they are not truly generalised. In the most comprehensive study to date, a size-based paramaterisation of the Rosenzweig-MacArthur system places restrictions on the relationships between the exponents of each parameter [16]. However, this in turn limits the types of perturbations that may be applied or investigated. Finally, there are discrepancies in the treatment of the functional response term between the theoretical and empirical literature. The theoretical literature broadly assumes that the limit of maximal consumption ties predator production to the prey’s and thus scales negatively to match prey production, however, there is empirical biological evidence for positive scaling [6].

Here, we present an alternate approach of paramaterising size-based predator-prey interactions for the classical Rosenzweig-MacArthur differential equations. Under this framework, we use multiple forms of analysis to create a robust picture of parameter sensitivity and required ranges for species coexistence in the context of real-world observations. We are able to show that, despite the number of assumptions inherent within this style of modelling, the mathematical restrictions are closely related to biological observations. Finally, we describe the conditions required to create an entirely size-invariant model, and show how empirically derived parameters generate ODE solutions that match real-world size-abundance distributions.

## Methods

### Parameterisation of the model

We begin with the Rosenzweig-MacArthur ODEs [24] and Holling II functional response,

$$\begin{array}{c} \frac{dR}{dt}=rR\left(1-\frac{R}{K}\right)-\frac{bR}{1+hbR}C\\ \frac{dC}{dt}=\epsilon \frac{bR}{1+hbR}C-\delta C. \end{array}$$ (1)

We have variables *R* for resources and *C* for consumers. The parameters *r* and *δ* are birth and death rates respectively. Carrying capacity is given by *K*, interaction rate *b*, handling time *h* and the conversion efficiency *ϵ*. To investigate the system across the full size range we scale the parameters by mass. Organism size (in g) is given by *S*_*R*_ for resources and *S*_*C*_ for consumers. We depart from [16] by constructing the functional response term in line with parameterisations used within experimental research [6, 25, 26]. Hence, the strictly positive parameters are expressed as

$$\begin{array}{c} r=r_{0}S_{R}^{\alpha_{r}}\\ K=K_{0}S_{R}^{\alpha_{K}}\\ b=b_{0}S_{C}^{\alpha_{b}}\\ h=h_{0}S_{R}^{\alpha_{hR}}S_{C}^{\alpha_{hC}}\\ \delta =d_{0}S_{C}^{\alpha_{\delta }}, \end{array}$$ (2)

where for each parameter *i*, the coefficients *i*_0_ may be standardised (Appendix A in S1 Appendix), and *α*_*i*_ denotes the scaling exponent. Next, we define the prey-predator mass ratio as *ρ*, where *ρ* > 0. We may then relabel the parameters *r*, *h* and *K* in terms of the consumer,

$$\begin{array}{c} \hat{r}=r_{0}{\left(\rho S_{C}\right)}^{\alpha_{r}}=r_{0}\rho^{\alpha_{r}}S_{C}^{\alpha_{r}}\\ \hat{h}=h_{0}{\left(\rho S_{C}\right)}^{\alpha_{hR}}S_{C}^{\alpha_{hC}}=h_{0}\rho^{\alpha_{hR}}S_{C}^{\alpha_{hR}+\alpha_{hC}}\\ \;\;\;\;\;\;\;=h_{0}\rho^{\alpha_{hR}}S_{C}^{\alpha_{h}}\\ \hat{K}=K_{0}{\left(\rho S_{C}\right)}^{\alpha_{K}}=K_{0}\rho^{\alpha_{K}}S_{C}^{\alpha_{K}}. \end{array}$$ (3)

With this approach we extend the results of [16] by placing no restrictions on the exponents, allowing *h* to be an independent term which may be matched to empirical observations. We now also follow standard practice by setting *ϵ* ∝ *ρ*, that is, the conversion efficiency is proportional to the prey-predator mass ratio [16]. Next, we use a standard rescaling of (1) to reduce the number of parameters and simplify analyses. We set $\tilde{R}=1/\left(b\hat{h}\right)$, $\tilde{C}$ = $\epsilon /(b\hat{h})$, $\mu =\hat{K}b\hat{h}$, and define $u=R/\tilde{R}$ and $v=C/\tilde{C}$. After scaling time by $\hat{r}$ such that $t=\hat{r}s$, and defining $\gamma =\epsilon /(\hat{h}\hat{r})$ and $\omega =\delta /\hat{r}$, we arrive at the new system

$$\begin{array}{c} \frac{du}{ds}=u\left(1-\frac{u}{\mu }\right)-\frac{\gamma uv}{1+u}\\ \frac{dv}{ds}=\frac{\gamma uv}{1+u}-\omega v. \end{array}$$ (4)

The parameters in (4) are also all strictly positive and scale across the size range. For completeness, we provide the explicit relationship between the old and new parameters in Table 1, and the system of equations with substituted terms is

$$\begin{array}{c} \frac{du}{ds}=u\left(1-\frac{S_{C}^{-\alpha_{h}-\alpha_{b}-\alpha_{K}}}{K_{0}h_{0}b_{0}\rho^{\alpha_{K}+\alpha_{hR}}}u\right)-\frac{\epsilon S_{C}^{-\alpha_{h}-\alpha_{r}}}{h_{0}r_{0}\rho^{\alpha_{r}+\alpha_{hR}}}\frac{uv}{1+u}\\ \frac{dv}{ds}=\frac{\epsilon S_{C}^{-\alpha_{h}-\alpha_{r}}}{h_{0}r_{0}\rho^{\alpha_{r}+\alpha_{hR}}}\frac{uv}{1+u}-\frac{\delta_{0}S_{C}^{\alpha_{\delta }-\alpha_{r}}}{r_{0}\rho^{\alpha_{r}}}v. \end{array}$$ (5)

**Table 1:** Relationship between parameters in original and rescaled Rosenzweig-MacArthur system. Here, *α*_*h*_ = *α*_*hR*_ + *α*_*hC*_.

	Definition	Coefficient	Exponent
*μ*	$\hat{K}\hat{h}b$	$\mu_{0}=K_{0}h_{0}b_{0}\rho^{\alpha_{K}+\alpha_{hR}}$	*α*_*μ*_ = *α*_*K*_s + *α*_*h*_ + *α*_*b*_
*γ*	$\epsilon /\hat{h}\hat{r}$	$\gamma_{0}=\epsilon /\left(r_{0}h_{0}\rho^{\alpha_{r}+\alpha_{hR}}\right)$	*α*_*γ*_ = −*α*_*h*_ − *α*_*r*_
*ω*	$\delta /\hat{r}$	$\omega_{0}=\delta_{0}/\left(r_{0}\rho^{\alpha_{r}}\right)$	*α*_*ω*_ = *α*_*δ*_ − *α*_*r*_

The expression *S*_*R*_ = *ρS*_*C*_ facilitates interpretability in downstream analyses. All exponent terms may be collected within *S*_*C*_, which we henceforth refer to as *S*. This provides simplified expressions within Table 1 and (5), yet we may still examine the impacts of perturbations to any one parameter. Table 2 summarises parameter exponent ranges within empirical research. Code used to generate the following results and figures is available at https://github.com/jcmckerral/universalallometry.

**Table 2:** Literature bounds on parameter values. The top portion of the table outlines scalars. The second block summarises scaling exponents, and the third block the full exponent range for Eq (4) where extreme min-max limits are given for completeness. However, there is consensus that *α*_*r*_, *α*_*δ*_ ≃ −1/4, also verified in a substantial recent review [27]. Similarly, despite the potential range for *α*_*b*_ and *α*_*h*_, in large generalised studies typically 1/2 ≤ *α*_*b*_ ≤ 1 [25, 26], and *α*_*h*_ ≤ 1/8 [6, 25, 26] which significantly constrain the exponent ranges in (4). The bottom section of the table therefore provides exponent values in the rescaled system based on upper/lower bounds for the most plausible generalised empirical scaling values for (2) (as determined by cited articles), i.e. those found from studies with large quantities of data, across broad taxonomic and size ranges, and/or named outliers being excluded [6, 22, 25–29].

Symbol	Parameter	Minimum	Maximum	References
*ρ*	Prey-predator mass ratio	1E-4	1E2	
*S*	(Consumer) mass, g	1E-10	1E7	
*ϵ*	Conversion efficiency	0	*ρ*	
*α*_*r*_	Birth rate	-0.81	-0.25	[6, 15, 16, 22, 27–31]
*α*_*δ*_	Death rate	-0.35	-0.22	[6, 15, 16, 22, 27–31]
*α*_*b*_	Interaction rate	-0.25	1.58	[6, 17, 25, 26, 29]
*α*_*K*_	Carrying capacity	-0.88	-0.74	[6, 16, 31]
*α*_*hR*_	Handling time (resource)	0	1	[15, 25, 26]
*α*_*hC*_	Handling time (consumer)	-1.1	0	[6, 15, 25, 26]
*α*_*μ*_	(maximal range)	-2.2	1.84	
*α*_*γ*_	(maximal range)	-0.75	1.92	
*α*_*ω*_	(maximal range)	-0.1	0.59	
*α*_*μ*_	(likely range)	-1.38	0.18	[6, 25, 26, 29]
*α*_*γ*_	(likely range)	0	1.25	[6, 25–27]
*α*_*ω*_	(likely range)	-0.05	0.05	[22, 27, 28]

## Results and discussion

### Coexistence & sensitivity

The non-trivial equilibrium of interest (coexistence) is obtained by equating the right side of (4) to zero and solving for *u* = u* and *v* = v*, yielding

$$\begin{array}{c} u^{*}=\frac{\omega }{\gamma -\omega }\\ v^{*}=\frac{\mu \gamma -\mu \omega -\omega }{\mu {\left(\gamma -\omega \right)}^{2}}. \end{array}$$ (6)

For there to be non-negative values for (u*, v*), we require that

$$\begin{array}{c} \gamma >\omega \;\text{or}\;\frac{\gamma }{\omega }>1 \end{array}$$ (7a)

and

$$\begin{array}{c} \mu >\frac{\omega }{\gamma -\omega }. \end{array}$$ (7b)

For the Jacobian of the right side of (4) evaluated at the equilibrium point (u*, v*), the condition *det* > 0 is also fulfilled by (7b). We may express (7b) as *γ*/*ω* > 1+ 1/*μ*. As all parameters are strictly positive, if (7b) is satisfied, it immediately follows that (7a) is satisfied also. The inequality

$$\begin{array}{c} \frac{\gamma }{\omega }<\frac{\mu +1}{\mu -1} \end{array}$$ (8)

determines the sign of the trace of the aforementioned Jacobian, which dictates whether the system converges to a point or to a stable limit cycle, and there is a Hopf bifurcation at equality. The dynamical characteristics of the Rosenzweig-MacArthur system have been explored in depth elsewhere (such as [24, 32, 33] and references within). Our focus is the interplay between biological and mathematical constraints. We now interrogate the behaviour of the system and inequalities (7) and (8) using a series of complementary analyses, and discuss the mathematical implications in the context of empirical observations.

#### Handling time

The condition from (7a) is equivalent to

$$\begin{array}{c} \frac{\epsilon }{h_{0}\delta_{0}S^{\alpha_{h}+\alpha_{\delta }}}>1. \end{array}$$ (9)

When investigating coexistence across the large size ranges considered in this study (>15 orders of magnitude), the coefficients on the left hand side of this inequality will have negligible impact relative to the scaling exponents of the parameters, as in log space they may translate the intercept of the defined line but cannot change its slope. Therefore, we instead examine the exponents given in (9) and observe that if *α*_*δ*_ + *α*_*h*_ < 0 the condition may fail for small organisms. As *α*_*δ*_ is tightly constrained (Table 2, and comprehensively reviewed in [27]), to better understand the contribution of handling time to coexistence constraints, we now investigate system behaviour when varying the scaling of *h*, under the assumption that other exponents are either fixed or undergo only minor perturbations; their relative contributions to coexistence properties are explored in later sections.

Handling time’s classical null model from Yodzis & Innes [15] based on metabolic theory is equivalent to $h\propto S_{R}^{1}S_{C}^{-3/4}$, or $\hat{h}\propto S^{1/4}$ as derived in [25]. A null model derivation assumes the maximal consumption rate of the predator—the inverse of handling time—scales with metabolic demand $S_{C}^{3/4}$, and the per-prey metabolic demand is therefore $S_{C}^{3/4}S_{R}^{-1}$. This matches the assumptions of [16, 17], where the birth rate of the predator in the presence of unlimited resources is assumed to scale with the birth rate of prey, though it should be noted that other interpretations of the same model either do not normalise against prey mass e.g. [31] or do so implicitly e.g. [6]. However, consideration of physiological traits’ nuanced impact on maximal consumption, and equivalently handling time, has since led to a departure from the traditional metabolic framework. Attacking, killing, then eating and digesting prey all impact the parameter *h* [26] and the prey’s contribution to the process should be incorporated [25, 26]. Handling time has therefore been recast to the more biologically representative form we use in this study: $h\propto S_{R}^{\alpha_{hR}}S_{C}^{\alpha_{hC}}$, where typically 0 ≤ *α*_*hR*_ ≤ 1 and −1 ≤ *α*_*hC*_ ≤ 0 [25, 26]. The exponents *α*_*hR*_, *α*_*hC*_ have been empirically determined in several reviews and display considerable variability [6, 25, 26, 34]. We now discuss the implications this variability has for coexistence under the inequalities in (7a) and (7b).

**Fig 1:**
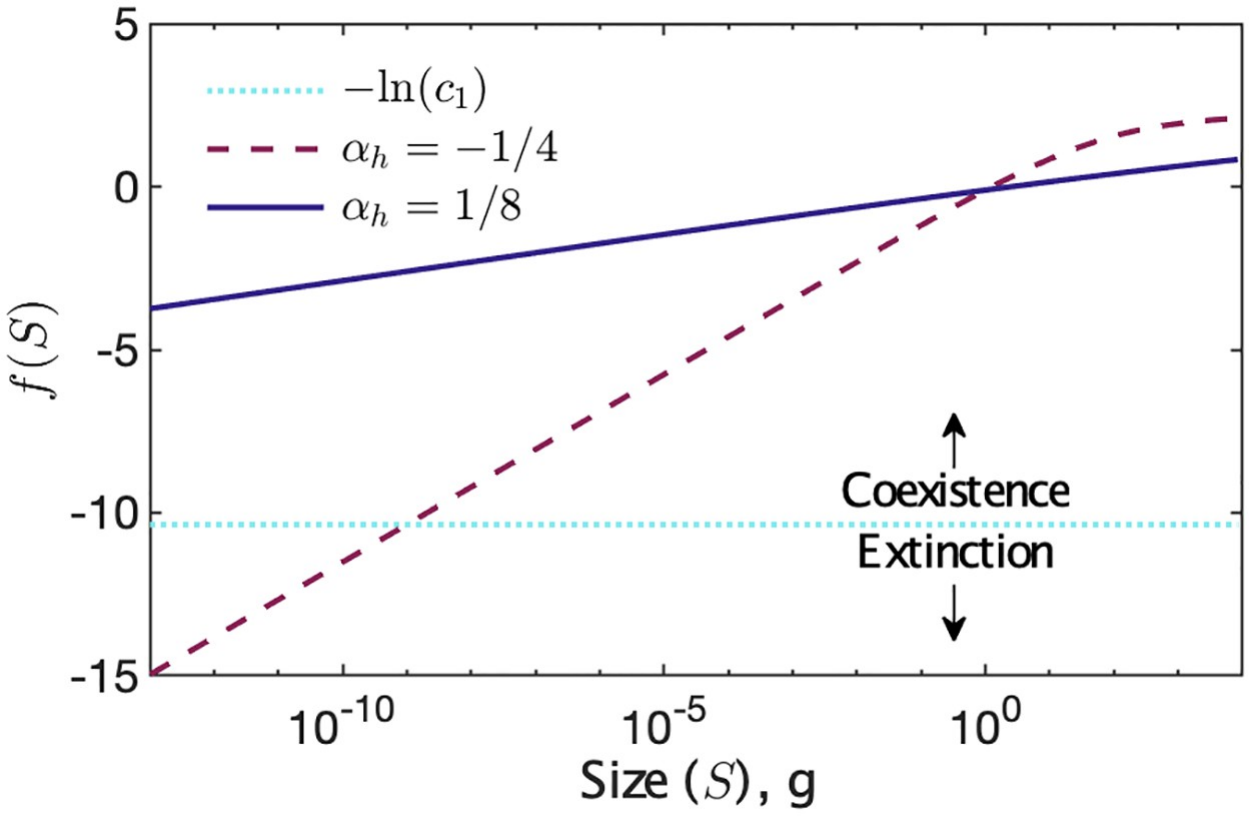
Graphical representation of coexistence condition (7b). Rearranging (10): $\text{ln}\left(S^{-\alpha_{h}-\alpha_{\delta }}\right)-\text{ln}\left(1+c_{2}S^{-\alpha_{K}-\alpha_{h}-\alpha_{b}}\right)>-\text{ln}\left(c_{1}\right)$. We denote the left side of the inequality as *f*(*S*); if *f*(*S*) > −*ln*(*c*_1_), there is coexistence. Under the feasible values in Table 2, here *α*_*K*_ and *α*_*b*_ have less effect than *α*_*h*_; we thus set *α*_*K*_ = −3/4 and *α*_*b*_ = 1/2.

Two of the three reviews listed above conclude that the predator-prey components of handling time scale more gently (whether positive, or negative) than null models predict. However, the resultant exponent for $\hat{h}$ is positive (≃1/3) for arthropod functional responses [25], but negative (≃−1/8) when examining broader taxonomic groups [26]. Most of the organisms in [26] display negative scaling for $\hat{h}$ in taxa-specific breakdowns due to gentler scaling of the resource exponent. Only *α*_*hC*_ is assessed in [6], which is calculated across a wide range of taxa for 2D and 3D environments and for a larger mass range than in [26]; for the 2D and 3D case *α*_*hC*_ ≃ −1.1. The authors account for the steeper scaling relative to metabolic expectation by noting that feeding is an active process scaling with maximal rather than basal metabolism [6]. To calculate the scaling of $\hat{h}$ from the empirical assessment in [6], we use the assumption *α*_*hR*_ = 1, which is the parameter’s upper limit. This implies the exponent *α*_*h*_ ≤ −0.1, and that $\hat{h}$ scales below the value of 1/4 assumed by previous theoretical work on the model. Conceptually, this indicates that the parameter may be constrained by physical processes rather than a bioenergetic flux balance. Note that despite the phenomenological formulation of the functional response predator production is implicitly constrained by the prey density. We next examine coexistence condition (7b), which may be expressed as *γ*/*ω* > 1 + 1/*μ* meaning ln(*γ*/*ω*) > ln(1 + *μ*^−1^); if we then substitute the original parameters we arrive at the inequality

$$\begin{array}{c} \text{ln}\left(c_{1}S^{\alpha_{h}-\alpha_{\delta }}\right)>\text{ln}\left(1+c_{2}S^{-\alpha_{K}-\alpha_{h}-\alpha_{b}}\right), \end{array}$$ (10)

where *c*_1_, *c*_2_ are constants derived from the coefficients. Considering (10) together with the empirical behaviour of $\hat{h}$, if *α*_*h*_ ≃ −1/5 or less across the size range, smaller organisms may violate this condition (Fig 1).

Similar to the behaviour of the first inequality (9) in this section, significantly perturbing the coefficients does not qualitatively change this behaviour. For example, a change of 3 or 4 orders of magnitude in the coefficient ratios will result in a vertical translation of the line ln(*c*_1_) of 3 to 4, which may impact results if one were to consider the point of intersection with *f*(*S*) for restricted size ranges (e.g. within restricted taxonomic groups), but do not affect the global behaviours of the complete size distribution considered in this study. In addition, even if the coefficients were to vary by an arbitrarily large amount, should the variation remain proportionally consistent, the ratios would remain constant; that is, the position of the coexistence line would not change. Conversely, Fig 1 shows that a relatively small perturbation of 0.6 in *α*_*h*_ alters the value of the term *f*(*S*) by over 10 across the size range, with a particularly significant impact on the smallest organisms. Therefore, provided that *α*_*h*_ ≃ −*α*_*δ*_ then all sizes will fulfil the condition, allowing coexistence across the full span of the model. There is empirical support for these observations. The taxonomic group breakdowns in [26] indicate that smaller taxa may display positive scaling for $\hat{h}$ as concluded in [25] and the strongly negative scaling is observed in macro-organisms, particularly vertebrates. The notable exception of unicellular marine organisms (*α*_*h*_ ≃ −1/3) has a very small sample size; further experimental studies and especially those which generate larger data sets may potentially reach alternate conclusions and we leave this for future work.

Next, we examine the contributions of the carrying capacity and interaction rate parameters *K* and *b* to coexistence and dynamical properties of the model.

#### Carrying capacity and scaling of population cycling

As the right side of (7b) is equal to u*, the coexistence condition may also be expressed as

$$\begin{array}{c} \mu_{0}S^{\alpha_{\mu }}=\mu_{0}S^{\alpha_{K}+\alpha_{h}+\alpha_{b}}>u^{*}, \end{array}$$ (11)

where $u^{*}\propto S^{\alpha_{\delta }+\alpha_{h}}$. If we combine (7b) and (8), we obtain

$$\begin{array}{c} \mu_{0}S^{\alpha_{\mu }}=\mu_{0}S^{\alpha_{K}+\alpha_{h}+\alpha_{b}}>1. \end{array}$$ (12)

Together, (11) and (12) indicate the carrying capacity must be sufficiently high for a sustainable prey population, and that predator attack rates must be high enough to compensate for mortality across all sizes. Assuming reasonable values for *α*_*K*_, *α*_*b*_ such as those given in the body and caption of Table 2 respectively, these inequalities will generally hold. Whilst the lower bound of *α*_*b*_ ≃ 1/2, estimates of the ‘universal’ value from comprehensive reviews suggest a number in the range 0.6 < *α*_*b*_ < 0.9 [25, 26]. This concurs with the properties of inequality (12), which—even if worst-case values are set for *K* and *h*—allows for coexistence across the full size range provided *α*_*b*_ does not exceed 0.9 by a significant margin, or equivalently, *α*_*μ*_ does not exceed ≃0.2. Smaller (or even negative) values of *α*_*b*_ serve to improve coexistence properties, suggesting that generalised empirical values for *α*_*b*_ conform well to the mathematical properties of the model, sitting at an upper bound of ≃0.9. Under the original system (1) and assuming coexistence, resource equilibria will scale with size as $R^{*}\propto S^{\alpha_{\delta }-\alpha_{b}}$ and consumer equilibria will scale as $C^{*}\propto S^{\alpha_{r}-\alpha_{b}}$. Despite their importance for the coexistence domain, the carrying capacity and half saturation do not meaningfully impact equilibria abundances in allometric paramaterisations. However, they do impact some properties of the limit cycle.

**Fig 2:**
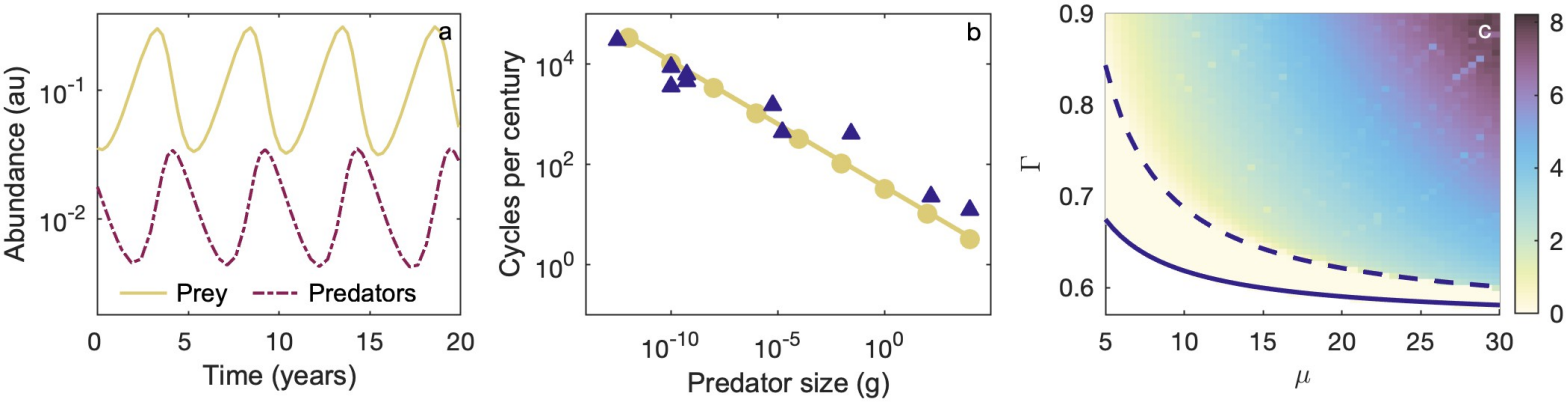
Properties of the limit cycle. Unless stated otherwise, scaling parameters are *α*_*K*_ = -3/4, *α*_*r*_ = *α*_*d*_ = −1/4, *α*_*b*_ = 1/2, and *α*_*h*_ = −1/8. (a) Predator (purple)-prey (yellow) oscillations for a 10g predator (b) Limit cycle period scaling for numerical (circles), empirical (triangles) and analytic (solid line) results. Only predators are shown. Data is from population study time series [17, 40–47]; periods were calculated by using supplied data files or extracting data from figures [48], and averaging time between peaks. (a)-(b) use empirical scaling of $\hat{h}$, where *α*_*h*_ = −1/8. (c) Dynamics of the rescaled system under different parameter values (i.e. all exponents are perturbed). Here, Γ = *γ*/*ω*, which is plotted against *μ*. The region below the solid line indicates no coexistence, between the solid and dashed lines denotes a sink to the equilibrium point, and above the dashed line a stable limit cycle. Colour indicates the difference between the (log) maximum and minimum predator abundances.

Allometric settings of the Rosenzweig-MacArthur system usually result in oscillating solutions due to size-scaled parameter values relative to the constraints in (8) [28]. Using data extracted from the literature for a broad range of taxa (Appendix A in S1 Appendix, Fig 2b), we find stronger empirical support than in previous work for a *S*^1/4^ scaling signal for the period *τ*_*t*_ [28], noting that *τ*_*t*_ indicates that we are considering period under the timescale of variable *t*. Expressions for theoretical scaling whereby $\tau_{t}\propto {\left(S^{\alpha_{\delta }+\alpha_{r}}\right)}^{-1/2}$ have previously been given in the literature [15, 28] and an exact equation for *τ*_*t*_ in [16], but to our knowledge a complete derivation for the equation in [16] has not previously been published. For the reader’s interest we provide one in Appendix B in S1 Appendix, where we show that, under the assumption that *S*_*C*_ ∝ *S*_*R*_,

$$\begin{array}{c} \tau_{t}=2\pi \sqrt{\frac{\epsilon /(\hat{h}\delta )+1}{\hat{r}\delta \left(\epsilon /\left(\hat{h}\delta \right)-1\right)}}, \end{array}$$ (13)

matching the findings of [16]. We find the theory agrees well with observed values, as our empirical data has a relationship $\tau_{t}^{-1}\propto S^{-0.2}$ (Fig 2b). Whilst multiple factors are proposed to affect cycle behaviour, these factors are made up of internal and external effects [17, 35, 36]. In particular, cycle periods are proposed to be predominantly driven by internal physiology such as maternal generation time, whereas cycle amplitude is thought to be dominated by external effects such as predation or other environmental influences [37]. With some caveats due to a temperature dependency and clear contribution of life history traits, at a broad scale maternal effects (i.e. generation times) are suggested to mirror the inverse of the maximal growth rates *r*, that is, the period should fall close to *S*^1/4^ as predicted by the model [35, 38, 39]. The empirical evidence for 1/4 power scaling is particularly strong for mammals [28, 35, 37, 39]. We note that most prior work has been restricted to specific taxonomic groups or across smaller size ranges than we consider here, as we include prokaryotes, protists, invertebrates as well as herbivorous and carnivorous mammals in our dataset (Appendix A in S1 Appendix). The notable exception of [38], with a generously sized and wide-ranging dataset, finds a slightly steeper scaling of *τ*_*t*_ ∝ *S*^0.31^ across aggregated taxa groups. However, the within-group exponents (considering insects, zooplankton/protists, and vertebrates independently) are shallower at ≃0.2 on average, which may suggest that there were challenges associated with aggregating data across different studies. That being said, period scaling of 0.2 to 0.3 across various taxa groups is within expected empirical variability, especially when studies have limited data, and we expect that the generation and synthesis of large, broad datasets may shed light on the validity of the *τ*_*t*_ ∝ *S*^1/4^ Rosenzweig-MacArthur model prediction in future.

With respect to the cycle amplitudes, previous work has found that the ratio of maximum to minimum densities is size-invariant [28], indicating that the oscillation amplitude decreases with increasing size. Further qualitative support that this mathematical behaviour is aligned with features of real-world biological systems is given by the fact that log-transformed size-density relationships demonstrate near-constant variance across 15+ orders of magnitude [13, 27]. Under our paramaterisation the log-scaled oscillations are relatively sinusoidal and may be constrained between one to three orders of magnitude (Fig 2a). Hence, they are more realistic than allometric Lotka-Volterra dynamics which push predator populations to unreasonably low levels with fluctuations exceeding 15 orders of magnitude [17]. Unfortunately early efforts to find analytic approximations of the oscillation amplitude of the Rosenzweig-MacArthur system have not been generalised [49]. Recent results have only been derived for specific—and restricted—parameter values [50]. However, the rescaled system (4) provides scope for us to examine the effects of perturbations in a simplified manner. In Fig 2c, we show through simulation that perturbations to all parameters impact the magnitude of the fluctuation of the limit cycle. However, unless these perturbations are applied to *α*_*r*_ or *α*_*δ*_, the oscillation amplitude will, within a small noise factor, remain invariant with respect to the mean population density, which reflects empirical findings [28]. Ecological theory and observation suggest that oscillation amplitudes are impacted by a myriad of environmental factors, reflecting the fact that the fluctuation size in the system may be impacted by changes to any parameter. Thus, the qualitative behaviour of the Rosenzweig-MacArthur’s limit cycle better reflects known biology than the Lotka-Volterra system [17, 37]. We next use a sensitivity analysis to assess the system’s robustness to different forms of perturbation.

#### Sensitivity

A local sensitivity analysis provides a first-order approximation of the relative impact of changing parameters on the solutions of (4) near the system’s equilibria. We adhered to the methodology described in [51]. In order to check how sensitive the system, $\dot{x}$, is to small changes in parameters, λ_*i*_, we construct a sensitivity function, *S*(*t*), such that

$$\begin{array}{c} S\left(t\right)=\frac{\partial }{\partial \lambda }x\left(t,\lambda \right) \end{array}$$ (14)

and *x*(*t*, λ) is a solution of $\dot{x}$. Next, we characterise the solution to the sensitivity equation given by

$$\begin{array}{c} \dot{S}\left(t\right)=A\left(t,\lambda_{0}\right)S\left(t\right)+B\left(t,\lambda_{0}\right)\text{,}\;S\left(t_{0}\right)=0. \end{array}$$ (14)

**Fig 3:**
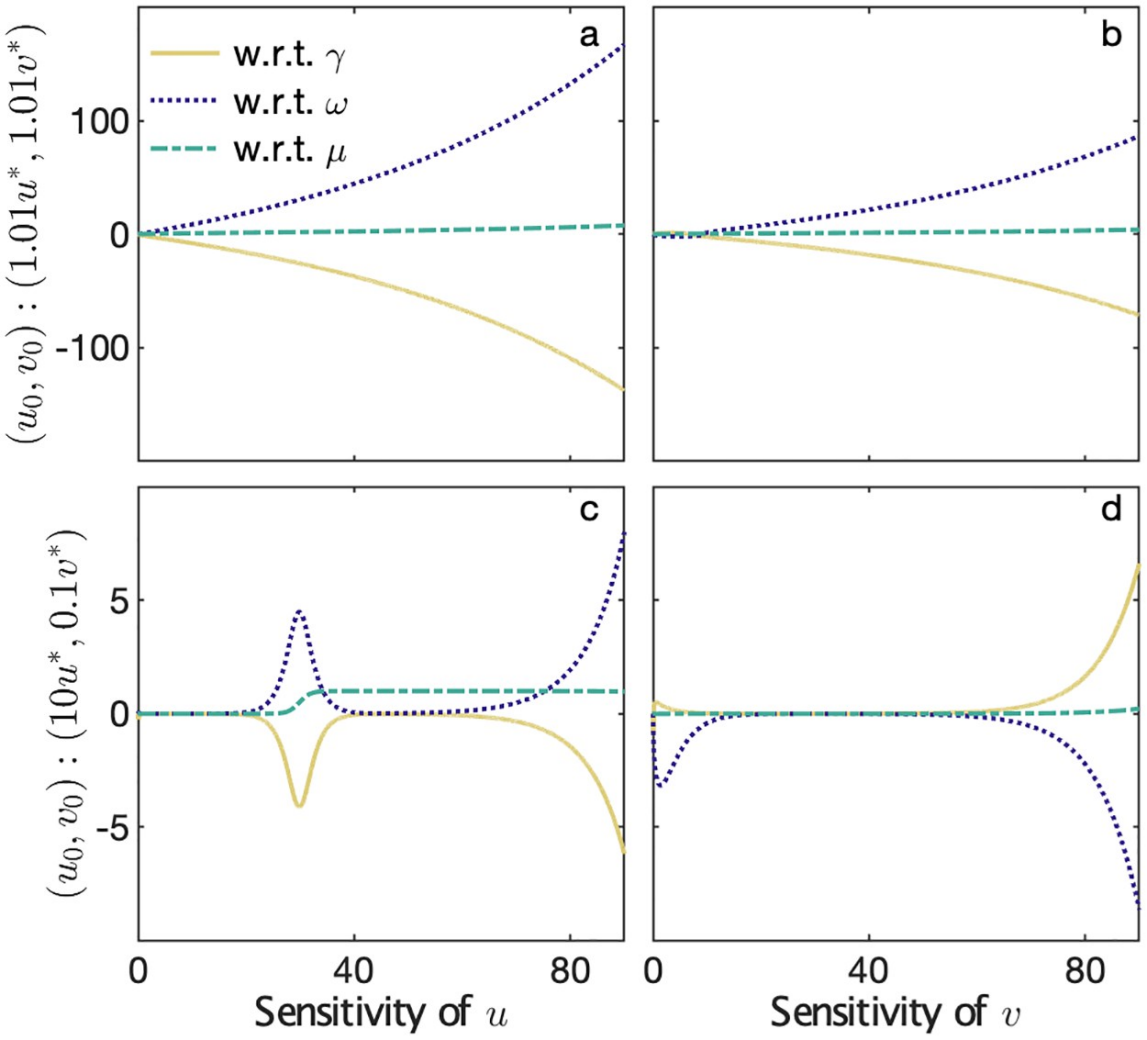
Sensitivity of rescaled system. *x*-axis denotes time (au). (a-b): Sensitivity of the solutions of (4) to perturbations to each of the parameters under an initial condition near the point (u*, v*). (c-d): As above, except under an initial condition (10u*, 0.1v*); the trajectory also converges to the limit cycle.

Applying (14) to (4), *A* is the Jacobian of (4) with respect to variables *u* and *v*, and *B* is the Jacobian of (4) with respect to parameters *μ*, *γ*, and *ω*, both of which are evaluated at nominal parameter values. After setting initial conditions *u*_0_ and *v*_0_, we obtain numerical solutions for (14). Fig 3 shows the trajectories for two initial conditions, noting that these are not the trajectories of the rescaled Rosenzweig-MacArthur system (4), but instead show the relative impacts of perturbing different parameters on the long term evolution of its solutions. Firstly, we show *u*_0_ and *v*_0_ in the neighborhood of u*, v* respectively (Fig 3a and 3b), and secondly for *u*_0_, *v*_0_ an order of magnitude greater/smaller than u*, v* respectively (Fig 3c and 3d). The qualitative behaviour remains the same in both cases. For an initial state near the equilibrium (Fig 3a and 3b), there is monotonic behaviour as the system converges to the limit cycle. In the case of Fig 3c and 3d the limit cycle emerges after some critical time *t*_*c*_. This analysis reflects the globally stable nature of the Rosenzweig-MacArthur equations and shows that the system is least sensitive to *μ*, providing further support that perturbations to *r* and *δ* have the largest potential impacts on its dynamics. The qualitative behaviour is similar for other nominal parameter values, provided they are not set on the other side of the bifurcation boundary. The exponents with the least empirical variation—by a significant margin—are *α*_*r*_ and *α*_*δ*_, mirroring the mathematical constraints. That is, the dynamical behaviour of the system is relatively robust to perturbing the functional response parameters displaying the highest empirical variance.

### Applications

The rescaled parameter definitions in Table 1 may be used to determine a size-invariant system by balancing the exponents. This is desirable as it is straightforward to set coexistence for an arbitrary size range: the rescaled system remains fixed, and transforming back to the original parameter space merely translates and stretches (or squashes) the dynamics in the base co-ordinates. This facilitates analytic study of the equations as it greatly simplifies the task at hand: only one equation (the size-invariant version of (4), which has constant parameters) needs be studied to understand the dynamical behaviour of (1) for any sized predator or prey. This is certainly mathematically expedient and we note that the majority of allometric studies to date have used this approach. To determine the scaling values necessary, the definition of $\omega =\delta /\hat{r}$ indicates that the size scaling of *r* must match *δ*, both of which consistently display an exponent of −1/4. It immediately follows that *α*_*h*_ = 1/4 as $\gamma =\epsilon /\hat{h}\hat{r}$. Assuming that carrying capacity scales with a −3/4 exponent, and using the definition $\mu =\hat{K}\hat{h}b$ it follows that *α*_*b*_ = 1/2. Indeed, previous analytic research into scaling dynamics of the Rosenzweig-MacArthur system places limitations around scaling values using similar principles, which reduces the complexity of the analysis but potentially decreases the model’s applicability to real-world systems [16]. In particular, given the tenuous empirical support for $\hat{h}\propto S^{1/4}$, it may be more biologically sound to assign a value where *α*_*h*_ ≤ 0; furthermore, our results from Sections 3.1.1 and 3.1.2 indicate that coexistence across the domain should be feasible under this condition. We find that it is still possible to generate a full size-abundance distribution when *α*_*h*_ ≤ 0. A recent size-density scaling analysis indicates that the relationship follows N* ∝ *S*^−1^ rather than the canonical N* ∝ *S*^−3/4^ (where N* is population density) [27], and we will show that we are able to reproduce this when choosing empirical scaling values across all parameters.

Having assessed coexistence properties in previous sections, we now note a possible extension to parameter *b* (interaction rate) and also consider whether our treatment of *ϵ* (conversion efficiency) is reasonable for the model. Whilst a static value in the range 0.6 < *α*_*b*_ < 0.9 is accepted as a reasonable generalised exponent based on major reviews [25, 26], a limitation of our treatment of *b* is that we place no restrictions on the interactions between a predator and any arbitrary-sized prey. Predator-prey interaction processes are complex and increasing evidence suggests they follow a ‘hump-shaped’ curve with the predator-prey mass ratio [25]. A natural extension to our model would be to introduce a term reliant on *ρ* to the parameter *b*, where $b=f\left(\rho ,b_{0}\right)S^{\alpha_{b}}$, and *f*(*ρ*, *b*_0_) is a function assigning probability of prey capture based on the prey-predator size ratio. While a form for *f*(*ρ*) has been proposed [16], it would be possible to use a function encoding a broader range of life history traits for the tradeoff of introducing more parameters. Relevant processes to consider may include habitat effects on foraging, prey refuges, and optimal size ratios, resulting in further constraints on coexistence [25, 26, 34]. However, as this would introduce additional complexity, for the purpose of this study we assign a constant value for the coefficent, meaning that interaction rates are consistent across different prey-predator mass ratios and the exponent *α*_*b*_ = 2/3.

**Fig 4:**
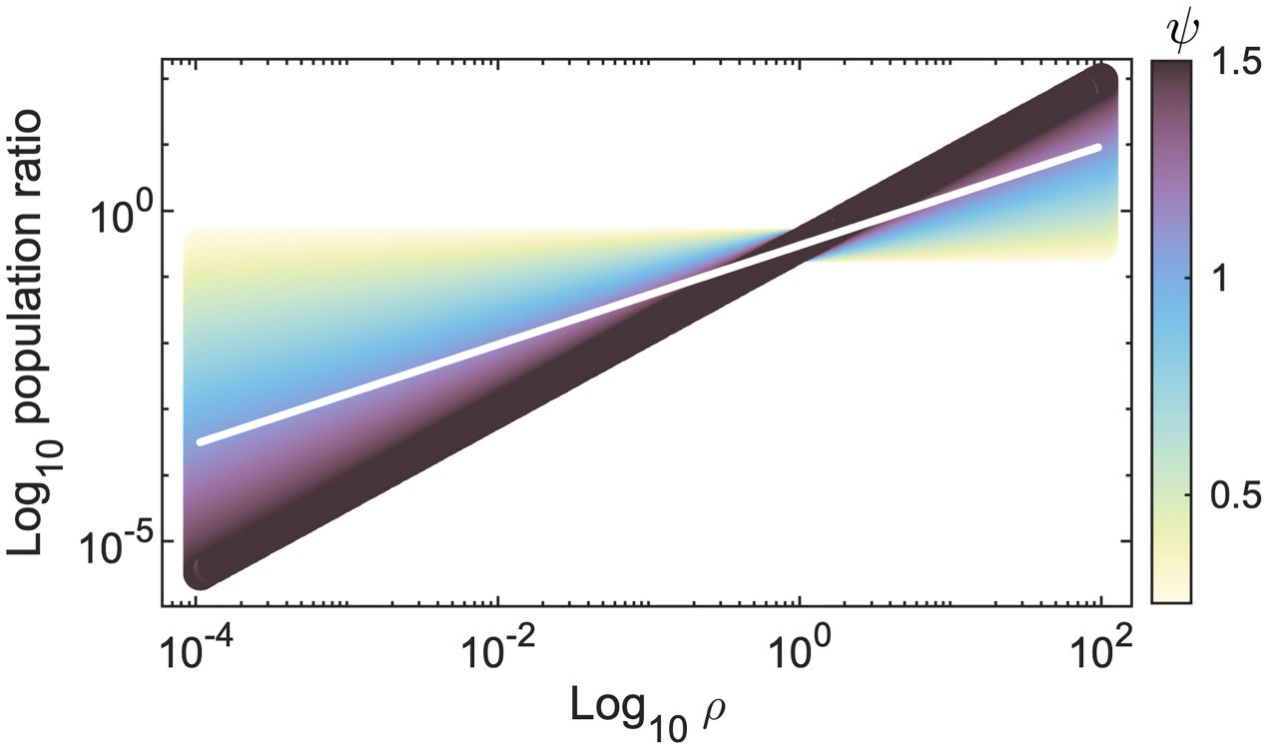
Impact of perturbing conversion efficiency *ϵ* by setting a function on *ρ*. Here, *ϵ* = *ρ*^*ψ*^, where −1/2 < *ψ* < 3/2. Colours map to the value of *ψ*, and the white line depicts *ψ* = 1. Y-axis shows the ratio of the predator-prey equilibria populations. Result is size invariant.

Our final consideration is the contribution of *ρ* to the conversion efficiency *ϵ*. The empirical distribution of *ρ* is approximately lognormal peaking at ≃0.02 [29]. Equilibria population ratios do not follow a 1:1 relationship with the size ratio of prey and predator when using the relationship *ϵ* = *ρ* (solid white line, Fig 4). For a fixed predator size and increasing prey size, organisms become less efficient at converting biomass. However, this result does not align with observed data. A review of 15,000+ predator-prey pairs concludes that size differences between predator and prey has an upper limit, potentially due to inefficiencies when the size discrepancy becomes too extreme [52]. Furthermore, the larger the predator, the more generalist its feeding strategies [25]; increases in prey biomass—which could indicate predators feeding on smaller prey—do not translate to a proportionate increase in predator biomass [53]. We therefore apply the assumption that energetic reward (and biomass conversion) for predator effort declines as the size difference increases, and that the scaling of *ρ* with equilibria population ratios is superlinear. We can implicitly capture the result by assigning a function *ϵ* = *aρ*^*ψ*^, where *ψ* is a scalar. For simplicity, we set *a* to 1, and in Fig 4, we assess the predator-prey population ratios for varying *ψ*.

A value of *ψ* > 1 increases the difference between the predator-prey populations; *ψ* < 1 reduces it. More sophisticated functional forms may include favourable size ratios or introduce a size dependency to the value of *ϵ*. However, there is limited empirical research on scaling properties of *ϵ* [13, 19, 30]. A theoretical investigation of optimal predator-prey size ratios together with more complex functional response formulations reflecting alternate foraging/feeding strategies may yield interesting results. We leave this question open for future work.

**Fig 5:**
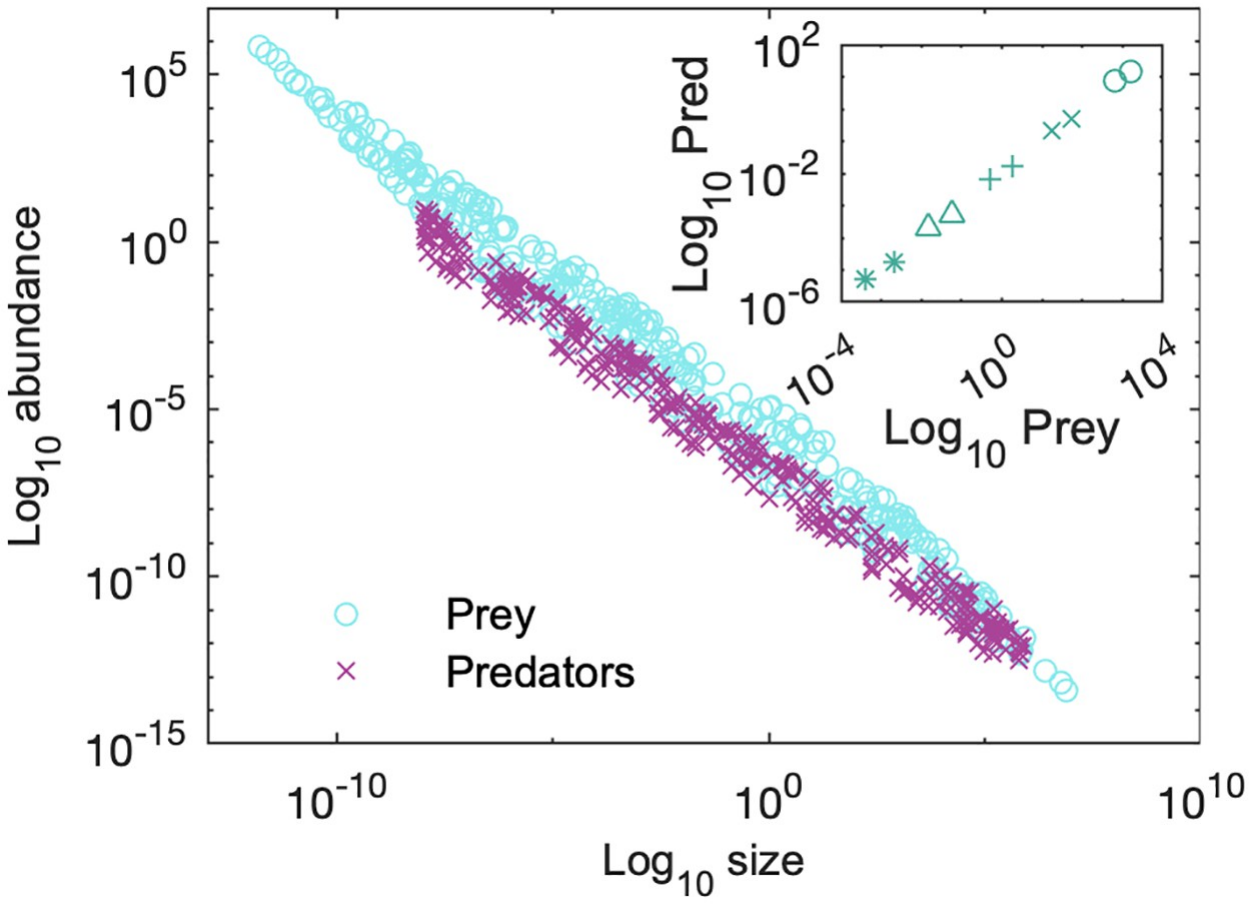
Main: Size-abundance data generated from the model. Circles depict prey abundances, and crosses predators. The parameter *ρ* was randomly selected within the interval of 1E-4 and 1E2. Parameter values are *ψ* ≃ 1.3, *α*_*r*_ = *α*_*δ*_ = −1/4, *α*_*h*_ = −0.1, *α*_*K*_ = −3/4 and *α*_*b*_ = 2/3. The full size-abundance distribution from the model scales to −0.95, matching the empirical distribution in [27] of −0.95. Inset: each pair of points are the maximum and minimum abundances attained by the predator/prey during limit cycle oscillations, under the same parameter values as for the main figure. We show examples of five predator sizes: 1E-6 (∘), 1E-4(×), 1E-2 (+), 1E0 (△), and 1E2g (*). For each *ρ* = 0.02. The slope within each symbol group ≃0.76. That is, increasing prey density does not result in a 1:1 increase in predator density. The scaling relationship is sublinear, matching the observations of [53].

To generate the size-abundance distribution shown in Fig 5, we use empirically motivated values where *ψ* ≃ 1.3, *α*_*r*_ = *α*_*δ*_ = −1/4, *α*_*h*_ = −0.1, *α*_*K*_ = −3/4 and *α*_*b*_ = 2/3. Coefficients are standardised to boundary values (Appendix A in S1 Appendix). The model’s distribution scales to −0.95, matching the value observed in [27]. The inset (generated from identical parameter values) shows the predator-prey density relationship, scaling at 0.76, close to the ≃3/4 findings in [53]. Our choice of *ρ* = 0.02 was motivated by the fact it is the most commonly observed prey-predator size ratio [52]. Changing the value of *ρ* will slowly perturb the predator-prey density slope due to *ψ* influencing the maximum and minimum values in the limit cycles in a nonlinear fashion (also seen in Fig 2c).

We propose that the interplay between scaling of *ρ* and predator-prey density slopes will have mathematically rich behaviours, especially as there is not yet a general solution for limit cycle amplitude in the Rosenzweig-MacArthur system [49, 50], but it is beyond the scope of this work to interrogate this question in detail. Our empirically-driven paramaterisation for our model generates a theoretical size-abundance distribution matching the largest data study to date [27], and we note that to generate Damuth’s law [12], simply changing the scaling of one parameter *b* such that *α*_*b*_ = 1/2 results in exponents of −0.81 and 3/4 for the size-abundance and predator-prey density scaling respectively. This sensitivity of the slope to interaction rate is reflected in biology. Variability in hunting and feeding strategies is a plausible reason that taxonomy-specific size-abundance distributions have exponents ranging from -1 to -1/4 as interaction rates are a critical driver for obtaining the energy for reproduction [12, 13, 18–20]. The functional response literature implies that the scaling of *b* is shallower for terrestrial endotherms than across broad taxonomic groups [26], which is precisely reflected by the model’s size-abundance scaling behaviour: Damuth’s original research focussed on mammals [12], yet Hatton *et al*.’s incorporates the full spectrum of eukaryotes [27]. We therefore suggest that the Rosenzweig-MacArthur system is suitable not only for large scale studies such as this, but that our results may be modified for subsets of taxa by using parameter scaling specific to those organisms, creating biologically meaningful models that may be used for prediction.

## Conclusions

Here, we investigate the links between empirical and theoretical allometric literature. The resource size dependency is eliminated from the system by explicitly encoding the prey-predator mass ratio, *ρ*. This simplifies analyses, allowing us to extend previous theoretical work by removing all restrictions on parameter relationships when examining properties of coexistence and macro scaling behaviours. Taxon-specific applications (which are far more sensitive to perturbations in coefficients due to the small size ranges under consideration) are likely to fall within the noise factor of the large size domains considered in this paper, however our methodology may still provide a parsimonious base for customising the equations in those settings. This may be useful for food web or trophic modelling, especially to mitigate against overfitting challenges [54]. Interrogating the model behaviour through three separate analyses provides a level of robustness to the finding that the mathematical constraints complement empirical observation. Contrary to most previous studies, we use an empirically determined parameterisation of the functional response term. Our results suggest that the standard approach of setting exponents based on metabolic theory may need to be reassessed [16]. The handling time parameter shows the greatest departure from those assumptions, and the highest variance, which is consistent with the massive trait variation in foraging strategies. Nevertheless, we find that results generated from an empirical setting agree with results in recent reviews of size-abundance scaling. This work may be extended in several ways. Firstly, one could incorporate temperature effects, for example after [22, 55], which may further stabilise the model by reducing the interaction strengths [30]. Secondly, additional empirical data on functional responses at the size extrema could more accurately define the scaling of $\hat{h}$ and *b*. Type I, Type III, or generalised functional responses may also be examined, although we note that system behaviour usually remains qualitatively similar over large size ranges [6, 31].

The broad limitation of allometry is that a generalist strategy can be a poor predictor of taxon-specific outcomes. Challenges to the framework arise not only from biological differences but also from physical or spatial processes, such as prey patchiness or heterogeneous habitat distribution [56]. Thus, care over interpretation and the applicability of results must be taken, particularly at the size limits in either direction. For example, prokaryotic reproduction rates fall between minutes and millenia [57, 58]. Furthermore, large organisms such as whales play a critical role in nutrient recycling; assuming a single species may be defined as a resource or consumer alone does not account for the intrinsic complexities within natural environments [59]. However, despite these caveats, allometric approaches have been found to outperform those explicitly encoding organisms’ individual and life-history traits when investigating a system’s macro properties [25]. Classical population dynamics models remain a powerful tool in ecology, and the consistency across many allometric laws suggest self-organising processes we are yet to unravel. We propose that systematically assessing where theoretical and empirical properties of allometric modelling diverge may assist in identifying plausible mechanisms governing these phenomena.

## Supporting information

S1 Appendix Supplementary data and derivations. Appendix A: Supplementary data, with tables of coefficient values used for the model and the empirical data used in Fig 2b. Appendix B: Derivation of the period of the limit cycle.
(PDF)
