# Supplementary material for: Empirical parameterisation and dynamical analysis of the allometric Rosenzweig-MacArthur equations
Source: PLoS One. 2023 Feb 27;18(2):e0279838. doi: 10.1371/journal.pone.0279838 (PMC9970096; doi:10.1371/journal.pone.0279838)
Supplement: S1 Appendix — Appendix A: Supplementary data, with tables of coefficient values used for the model and the empirical data used in Fig 2b. Appendix B: Derivation of the period of the limit cycle. (PDF) [file pone.0279838.s001.pdf]

S1 Appendix: Empirical parameterisation and dynamical analysis of the allometric Rosenzweig-MacArthur equations

Jody Fisher, Maria Kleshnina, Vladimir Ejov, Louise Bartle, James G. Mitchell, Jerzy A. Filar

Appendix A
Supplementary data

Table of coefficients

	Range in this study	Lower (literature)	Upper (literature)
r_0	1.00E-06 to 2.60E-06	3.70E-07	7.30E-06
d_0	4.00E-06	4.20E-08	5.60E-06
K_0	5.00E+00	5.62E-02	3.00E+02
h_0	3.00E-03 to 1.00E+01	1.00E-03	1.00E+01
b_0	1.00E-01	1.70E-02	9.49E+00

Table 1: Table showing coefficient values used in the study (based off empirical data). Coefficients are standardised against boundary values from the literature [3, 11, 9]. Rate measure units are in seconds and mass in grams.

Taxonomic data

Organism	Reference
Aedes aegypti	[13]
Alcaligenes faecalis IAM 1015	[12]
Azuki bean weevil	[14]
B. calyciflorus sensu strictu	[2]
Bosmina longirostris	[5]
Chlorella vulgaris	[2]
Colpidium campylum strain S	[12]
Daphnia galeata mendotae	[5]
Didinium nasutum	[10]
E. coli	[1]
Eotetranychus sexmaculatus	[6]
Heterospilus prosopidi (parasite)	[14]
Lepus americanus	[4]
Lynx canadensis	[4]
M. minutum	[2]
Paramecium aurelia	[10]
Toxorhynchites brevipalpis	[13]
Typhlodromus occidentalis	[6]

Table 2: Taxa included in the empirical data for limit cycle period (main text, Figure 2b), and references.

Appendix B
Limit cycle period

Background

We have the rescaled Rosenzweig-Macarthur system of equations,

$$\begin{aligned}\frac{du}{ds} &= u(1-\frac{u}{\mu}) - \frac{\gamma uv}{1+u} \\ \frac{dv}{ds} &= \frac{\gamma uv}{1+u} - \omega v,\end{aligned} \tag{1}$$

with strictly positive parameters μ, γ and ω.

The equality

$$\frac{\gamma}{\omega} = \frac{\mu+1}{\mu-1} \tag{2}$$

is the point of a Hopf bifurcation. Taking (2) instead into consideration as an inequality determines the sign of the trace of the system Jacobian, which therefore dictates whether the system converges to a point or to a stable limit cycle. We assume that $\frac{\gamma}{\omega} > \frac{\mu+1}{\mu-1}$, meaning that there is a globally stable limit cycle.

Deriving the period of the limit cycle

Previous work has provided analytic expressions for an approximation of the period of the limit cycle [15, 16], however, the explicit derivations were not given. Here, we provide a derivation for the analytic approximation of the limit cycle period. First, we note some particulars about well-known properties of the Rosenzweig-Macarthur system, namely that there are three stationary points, (u^*, v^*), $(0, 0)$, and $(\mu, 0)$, and that the limit cycle is globally stable.

To begin with, we wish to find a point near the limit cycle which is not the equilibrium point. To achieve this, we find the nullcline for u, that is

$$0 = u(1-\frac{u}{\mu}) - \frac{\gamma uv}{1+u}. \tag{3}$$

We have the trivial solution of $u = 0$, but also the two solutions

$$u = \frac{1}{2}(-1+\mu \pm (1+2\mu+\mu^2-4\gamma\mu v)^{1/2}). \tag{4}$$

Let us denote a chosen point along $u = \frac{1}{2}(-1+\mu+(1+2\mu+\mu^2-4\gamma v)^{1/2})$ as $\bar{u}$. As we assume that $\bar{u}$ is not the u-coordinate of an equilibrium point of the system, we may conclude that it must be the u-coordinate of another point $(\bar{u}, \bar{v})$ on the nullcline for u (Figure 1).

Furthermore, let us choose our point $(\bar{u}, \bar{v})$ such that $|\dot{v}|$ is maximal (minimal). Therefore, to find $|\dot{v}|$, we substitute $\bar{u}$ into the second equation of (1) such that

$$\begin{aligned}\frac{dv}{ds} &= \frac{\gamma \bar{u} v}{1+\bar{u}} - \omega v \\ &= \frac{\gamma\frac{1}{2}(-1+\mu+(1+2\mu+\mu^2-4\gamma\mu v)^{1/2})v}{1+\frac{1}{2}(-1+\mu+(1+2\mu+\mu^2-4\gamma\mu v)^{1/2})} - \omega v.\end{aligned} \tag{5}$$

As we have assumed $\bar{v}$ is where $|\dot{v}|$ is maximal (minimal), we may therefore find the value of v that maximises (minimises) the expression

$$\frac{\gamma\frac{1}{2}(-1+\mu+(1+2\mu+\mu^2-4\gamma\mu v)^{1/2})v}{1+\frac{1}{2}(-1+\mu+(1+2\mu+\mu^2-4\gamma\mu v)^{1/2})}, \tag{6}$$

as we are not on a stationary point and thus $|\dot{v}| \neq 0$. Computation in *Mathematica* (v 12.0.0) [7] provides a value of

$$\bar{v} = \frac{(1+\mu)^2}{4\gamma\mu}. \tag{7}$$

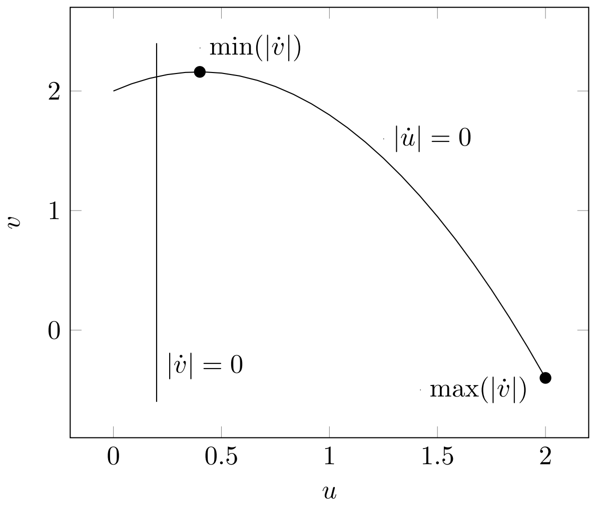

Figure 1: Schematic for the nullclines of (1), showing where $\dot{u} = \dot{v} = 0$, and points $(\bar{u}, \bar{v})$ where $|\dot{v}|$ is maximal, or minimal, under the assumption that $|\dot{v}| \neq 0$.

The period of the limit cycle may be approximated by

$$\tau = \frac{2\pi}{|Im(\lambda_i)|}, \tag{8}$$

where $|Im(\lambda_i)|$ is the imaginary part of the eigenvalues of the system's Jacobian [8, 16]. Thus, we evaluate the spectrum of the Jacobian of (1) at the point $(\bar{u}, \bar{v})$ and obtain

$$\lambda_i = \frac{1}{2\mu(\mu+1)} \left(\gamma\mu^2 - \gamma\mu - \omega\mu - \omega\mu^2 \pm i\sqrt{4\left(\gamma\mu^3 - \gamma\mu\right) - \left(\gamma\mu + \mu^2\omega + \mu\omega - \gamma\mu^2\right)^2} \right), \tag{9}$$

with the magnitude of the imaginary part of the eigenvalues being given by

$$|Im(\lambda_i)| = \frac{\sqrt{4\left(\gamma\mu^3 - \gamma\mu\right) - \left(\gamma\mu + \mu^2\omega + \mu\omega - \gamma\mu^2\right)^2}}{2\mu(\mu+1)}. \tag{10}$$

Next, we note that the approximation for the limit cycle period is taken near the bifurcation boundary. That is, we may consider the period of (1) in the neighborhood of the linearised system close to the parameter values at the bifurcation point. Therefore, we rearrange (2) to find the critical parameter value $\mu = (\gamma/\omega+1)/(\gamma/\omega-1)$, which we substitute into (10); after some algebra the equation reduces to

$$|Im(\lambda_i)| = \sqrt{\frac{\omega(\gamma - \omega)}{\gamma + \omega}}, \tag{11}$$

noting that in the presence of a limit cycle $\gamma > \omega$.

If we then substitute (11) into (8) to calculate the period τ, we have

$$\begin{aligned} \tau &= \frac{2\pi}{|Im(\lambda_i)|} \\ &= 2\pi\sqrt{\frac{\gamma + \omega}{\omega(\gamma - \omega)}}. \end{aligned} \tag{12}$$

Equivalence to prior results

Finally, we show how (12) may be mapped to Equation (28) in [15], which (expressed in the notation of our paper) is

$$\tau_t = 2\pi\sqrt{\frac{\epsilon/(\hat{h}\delta)+1}{\hat{r}\delta(\epsilon/(\hat{h}\delta)-1)}}, \tag{13}$$

and τ_t indicates that we are considering period under the timescale of variable t, and τ_s denotes that it is in the timescale of the rescaled system.

Recall that under our change of variables,

$$\mu = \hat{K}\hat{h}b \tag{14}$$
$$\gamma = \epsilon/(\hat{h}\hat{r})$$
$$\omega = \delta/\hat{r}$$
$$t = \hat{r}s. \tag{15}$$

Thus, as $\frac{dx}{dt} = \frac{dx}{ds}\frac{ds}{dt} = \frac{1}{\hat{r}}\frac{dx}{ds}$ we may write (13) as follows,

$$\begin{aligned}
\tau_t &= 2\pi\sqrt{\frac{\epsilon/(\hat{h}d)+1}{\hat{r}\delta(\epsilon/(\hat{h}\delta)-1)}} \qquad (16)\\
&= 2\pi\sqrt{\frac{(\gamma\hat{h}\hat{r})/(\hat{h}\delta)+1}{\hat{r}^2\omega((\gamma\hat{h}\hat{r})/(\hat{h}\delta)-1)}}, \text{ as } \hat{r}\omega = \delta \text{ and } \gamma\hat{h}\hat{r} = \epsilon,\\
&= 2\pi\sqrt{\frac{\gamma/\omega+1}{\hat{r}^2\omega(\gamma/\omega-1)}}, \text{ as we cancel } \hat{h} \text{ and } 1/\omega = \hat{r}/\delta\\
&= \frac{1}{\hat{r}}2\pi\sqrt{\frac{\gamma+\omega}{\omega(\gamma-\omega)}}, \text{ as } \hat{r} > 0,\\
&= \frac{1}{\hat{r}}\tau_s \text{ as required. } \square
\end{aligned}$$

References

[1] F. K. Balagaddé, H. Song, J. Ozaki, C. H. Collins, M. Barnet, F. H. Arnold, S. R. Quake, and L. You. A synthetic escherichia coli predator–prey ecosystem. *Molecular systems biology*, 4(1):187, 2008.

[2] B. Blasius, L. Rudolf, G. Weithoff, U. Gaedke, and G. F. Fussmann. Long-term cyclic persistence in an experimental predator–prey system. *Nature*, 577(7789):226–230, 2020.

[3] U. Brose, L. Cushing, E. L. Berlow, T. Jonsson, C. Banasek-Richter, L.-F. Bersier, J. L. Blanchard, T. Brey, S. R. Carpenter, M.-F. C. Blandenier, et al. Body sizes of consumers and their resources: Ecological archives e086-135. *Ecology*, 86(9):2545–2545, 2005.

[4] A. Eilersen and K. Sneppen. Applying allometric scaling to predator-prey systems. *Physical Review E*, 99 (2):022405, 2019.

[5] C. E. Goulden and L. L. Hornig. Population oscillations and energy reserves in planktonic cladocera and their consequences to competition. *Proceedings of the National Academy of Sciences*, 77(3):1716–1720, 1980.

[6] C. Huffaker et al. Experimental studies on predation: dispersion factors and predator-prey oscillations. *Hilgardia*, 27(14):343–383, 1958.

[7] W. R. Inc. Mathematica, Version 12.0.0. URL `https://www.wolfram.com/mathematica`. Champaign, IL, 2021.

[8] G. Iooss and D. D. Joseph. *Elementary stability and bifurcation theory.* Springer Science & Business Media, 2012.

[9] N. D. Lewis, M. N. Breckels, S. D. Archer, A. Morozov, J. W. Pitchford, M. Steinke, and E. A. Codling. Grazing-induced production of dms can stabilize food-web dynamics and promote the formation of phytoplankton blooms in a multitrophic plankton model. *Biogeochemistry*, 110(1):303–313, 2012.

[10] L. S. Luckinbill. Coexistence in laboratory populations of paramecium aurelia and its predator didinium nasutum. *Ecology*, 54(6):1320–1327, 1973.

[11] S. Pawar, A. I. Dell, and V. M. Savage. Dimensionality of consumer search space drives trophic interaction strengths. *Nature*, 486(7404):485–489, 2012.

[12] R. Sudo, K. Kobayashi, and S. Aiba. Some experiments and analysis of a predator-prey model: Interaction between colpidium campylum and alcaligenes faecalis in continuous and mixed culture. *Biotechnology and Bioengineering*, 17(2):167–184, 1975.

[13] M. Trpis. Interaction between the predator toxorhynchites brevipalpis and its prey aedes aegypti. *Bulletin of the World Health Organization*, 49(4):359, 1973.

[14] S. Utida. Cyclic fluctuations of population density intrinsic to the host-parasite system. *Ecology*, 38(3): 442–449, 1957.

[15] J. S. Weitz and S. A. Levin. Size and scaling of predator–prey dynamics. *Ecology letters*, 9(5):548–557, 2006.

[16] P. Yodzis and S. Innes. Body size and consumer-resource dynamics. *The American Naturalist*, 139(6): 1151–1175, 1992.
